# Supplementary material for: Qubit-mediated deterministic nonlinear gates for quantum oscillators
Source: Sci Rep. 2017 Sep 14;7:11536. doi: 10.1038/s41598-017-11353-3 (PMC5599512; doi:10.1038/s41598-017-11353-3)
Supplement: Supplementary file 1 — Supplementary Information [file 41598_2017_11353_MOESM1_ESM.pdf]

Supplemental material for Qubit-mediated deterministic nonlinear gates for quantum oscillators

Kimin Park,* Petr Marek, and Radim Filip
Palacky University, Czech Republic
(Dated: July 20, 2017)

PACS numbers:

I. SUPPLEMENTAL MATERIAL A: COMBINING TWO OPERATORS IN SU[2]

From properties of Pauli matrices which satisfy $\hat{\sigma}_i^2 = \hat{\mathbb{1}}$, we have $e^{i\vec{a}\cdot\vec{\sigma}} = \cos[\sqrt{\vec{a}\cdot\vec{a}}] + i(\vec{a}\cdot\vec{\sigma})\sin[\sqrt{\vec{a}\cdot\vec{a}}]/\sqrt{\vec{a}\cdot\vec{a}} \equiv \cos a + i(\vec{a}\cdot\vec{\sigma})\frac{\sin a}{a}$ for arbitrary vector form of operator $\vec{a}$, where $\vec{\sigma} = (\hat{\sigma}_x, \hat{\sigma}_y, \hat{\sigma}_z)$ is the Pauli vector and $a = \sqrt{\vec{a}\cdot\vec{a}}$ is a notational constant. Thus, we can use the formula $(\vec{a}\cdot\vec{\sigma})(\vec{b}\cdot\vec{\sigma}) = \vec{a}\cdot\vec{b} + i(\vec{a}\times\vec{b})\cdot\vec{\sigma}$ of SU[2] to combine the commuting operators $\vec{a}$ and $\vec{b}$ in vector form as

$$e^{i\vec{a}\cdot\vec{\sigma}}e^{i\vec{b}\cdot\vec{\sigma}} = \left(\cos a\cos b - \sin a\sin b\frac{\vec{a}\cdot\vec{b}}{ab}\right) + i\left(\cos a\sin b\;\frac{\vec{b}}{b} + \cos b\sin a\;\frac{\vec{a}}{a} - \sin a\sin b\frac{\vec{a}\times\vec{b}}{ab}\right)\cdot\vec{\sigma}. \quad (1)$$

The equation Eq. (1) in the main text was derived using the equation $\exp[\pm i\tau\hat{\sigma}_x\hat{A}]\exp[\pm i\tau\hat{\sigma}_y\hat{B}] = \cos[\tau\hat{A}]\cos[\tau\hat{B}] + i(\pm\cos[\tau\hat{B}]\sin[\tau\hat{A}]\sigma_x \pm \cos[\tau\hat{A}]\sin[\tau\hat{B}]\sigma_y - \sin[\tau\hat{A}]\sin[\tau\hat{B}]\sigma_z)$.

*Electronic address: park@optics.upol.cz
